# A framework and analytical exploration for a data-driven update of the Sequential Organ Failure Assessment (SOFA) score in sepsis^☆^

**DOI:** 10.1016/j.ccrj.2025.100105

**Published:** 2025-03-14

**Authors:** Drago Plečko, Nicolas Bennett, Ida-Fong Ukor, Niklas Rodemund, Ary Serpa-Neto, Peter Bühlmann

**Affiliations:** a Seminar for Statistics, Department of Mathematics, ETH Zürich, Switzerland; b Department of Anaesthesia and Perioperative Medicine, Monash Health, Melbourne, Australia; c Austin Hospital Intensive Care Unit, Melbourne, Australia; d Department of Anaesthesiology, Perioperative Medicine and Intensive Care Medicine, Paracelsus Medical University of Salzburg, Austria; e Australian and New Zealand Intensive Care Research Centre, School of Public Health and Preventative Medicine, Monash University, Melbourne, Australia

**Keywords:** Sepsis, Organ failure, Organ dysfunction, Suspected infection, Big data, Outcome prediction

## Abstract

**Objective:**

The Sepsis-3 consensus statement emphasised the need for data-based approaches to organ failure assessment and use the Sequential Organ Failure Assessment (SOFA) for this purpose. We aimed to develop a framework for a data-driven update to the SOFA score for patients with sepsis.

**Design:**

Systematic analysis of potential markers of organ dysfunction in a retrospective, observational study.

**Setting:**

Intensive care units from three tertiary hospital centres in the United States, the Netherlands, and Austria were included in the study.

**Participants:**

28 100 American, 5339 Dutch, and 2450 Austrian patients with suspected sepsis were included in this study.

**Measurements and main results:**

We assessed 56 organ function variables. We applied area under curve maximisation procedures to optimise the predictive power for mortality. We chose the most predictive biomarker for existing organ dysfunction domains and added a metabolic domain. We compared the area under the receiver operating characteristic curve and the area under the precision recall curve of the data-driven approach against the current SOFA system. The novel approach outperformed the current SOFA in all domains and databases (the area under the receiver operating characteristic curve: for US patients: 0.766 vs. 0.727, mortality: 10.7%; for Dutch patients: 0.70 vs. 0.653, mortality: 22.0%; for Austrian patients: 0.704 vs. 0.665, mortality: 22.0%; all p < 0.01 for the best performing score). The precision-recall curve confirmed such observations.

**Conclusions:**

We developed and validated a framework for a data-driven update to the SOFA to identify and classify organ dysfunction in suspected septic patients. This framework can be used to revise the SOFA score and its application to the identification and classification of sepsis.

## 1 Introduction

Sepsis remains one of the most challenging conditions in the intensive care unit (ICU) and the leading cause of mortality in the critically ill patients.^1^ The latest Sepsis-3 consensus definition highlighted the central role of organ dysfunction in the definition, identification, prognostication, and pathophysiological understanding of sepsis.^2^ Characterisation of such organ dysfunction in suspected septic patients (those with suspected infection and admitted to the ICU), however, continues to prove challenging and remains based on expert opinion.

In the Sepsis-3 definition, organ dysfunction was identified and quantified using a score developed according to expert opinion: the Sequential Organ Failure Assessment (SOFA) score.^3^ The SOFA score was chosen because it was historically well established, widely used, and relatively easily implemented. Moreover, although not originally designed as a predictive score, SOFA also performs reasonably well in the early prediction of outcome in ICU patients with infection.[4], [5], [6], [7], [8], [9], [10], [11], [12] The SOFA score, however, was developed in an era predating the advent of widespread arterial blood gas–linked lactate measurement, large digital epidemiologic datasets, and the statistical and computational techniques to handle such datasets. In contrast, new approaches to the definition of sepsis (as in Sepsis-3) have emphasised, but not yet delivered, on the need to apply a data-based approach to the identification, definitions, and classifications of organ dysfunction.^2^^,^^13^

Accordingly, we sought to explore, develop, validate, and test a data-based approach to identifying and defining the presence and severity of organ failure in patients with potential sepsis using three large critical care cohorts. We aimed to develop a framework for updating the SOFA score in a data-driven manner in order to deliver greater predictive performance in patients with suspected sepsis. Such a framework can be used by future consensus groups to guide their decision on how to develop a score that is simple, predictive, clinically significant, and robust.

## 2 Methods

### 2.1 Ethical approval

The Ethics Committee of Canton of Zürich waived the need for an ethical approval of the study (Request-2021-00678).

### 2.2 Study cohort

We studied patients from three large intensive care electronic health record (EHR) databases: the Medical Information Mart for Intensive Care (MIMIC-IV)^14^ database from the Beth Israel Deaconess Medical Center in Boston, Massachusetts, the Amsterdam University Medical Center (AUMC) database^15^ from the Department of Intensive Care Medicine of the Amsterdam University Medical Centre, and the Salzburg Intensive Care Database (SICdb)^16^ from the Department of Anaesthesiology and Intensive Care of the General Hospital Salzburg, Austria. From the three databases, all adult patients (aged >18 years) with suspected infection (who were at a risk of developing sepsis if organ failure occurred) within the first 2 days of ICU stay were included in our study cohort, whilst repeated ICU admissions were removed. Suspected infection was defined as the co-occurrence of antibiotic treatment and body fluid sampling.^4^ Following the approach of the original authors, body fluid sampling had to occur within 24 h of antibiotic treatment, whereas antibiotic treatment had to occur within 72 h after body fluid sampling^4^ for a patient to have a suspected infection.

All data used in our analyses are publicly available. The MIMIC-IV and SICdb datasets are available through Physionet,^17^ and the AUMC database is available through the Amsterdam Medical Data Science website.^18^ The For statistical analysis and data loading, we used the ricu R package^19^ and R Statistical Software^20^ (version 4.1.1). All the code used in the analyses is available on Github (https://github.com/eth-mds/dose). Throughout the manuscript, we report values for the MIMIC-IV dataset, with AUMC and SICdb values in brackets, unless otherwise explicitly stated.

### 2.3 Constructing the score

**Fig. 1:**
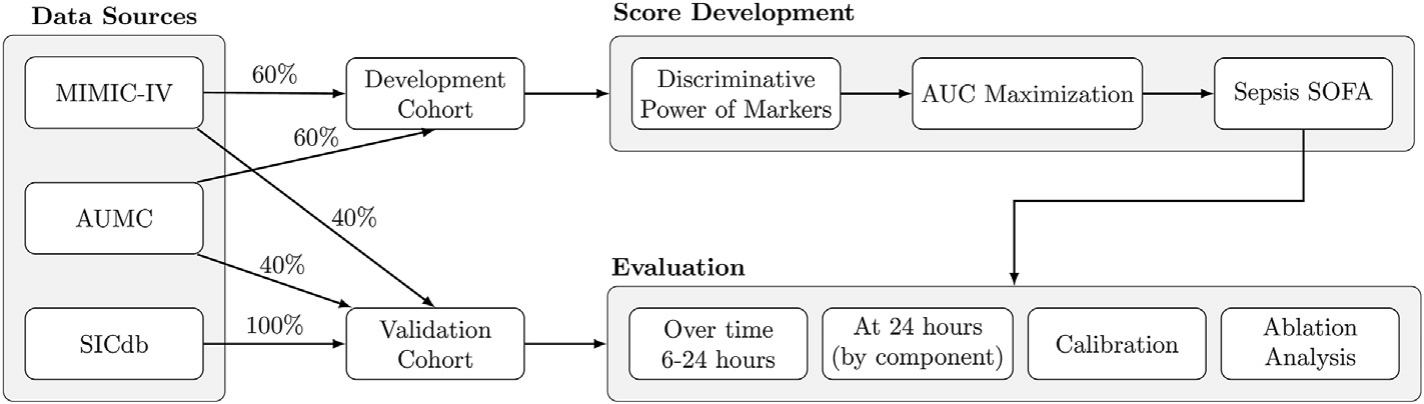
**Overview figure of the deployed framework.** Three datasets were used for developing and validating our predictive score (MIMIC-IV, AUMC, SICdb). MIMIC-IV and AUMC were split into 60% development and 40% validation cohorts, while SICdb was used only for validation. The development cohort was used to test the discriminative power of biomarkers, perform AUC maximisation, and construct the Sepsis SOFA score. The validation cohort was used for different evaluation analyses. AUMC: Amsterdam University Medical Center; ICU: intensive care unit; MIMIC-IV: Medical Information Mart for Intensive Care; SICdb: Salzburg Intensive Care Database; SOFA: Sequential Organ Failure Assessment.

The primary outcome of interest was in-hospital mortality. The steps of our data-driven framework are shown in Fig. 1.

Within our framework, 60% of the MIMIC-IV cohort (n = 26,104) and 60% of the AUMC cohort (n = 3253) were used (the development cohort). We started by determining a broad range of potential organ dysfunction biomarkers, which were reported across all the three databases (56 features in total). To assess the discriminative power of each biomarker, we determined its area under the receiver operating characteristic curve (AUROC) for mortality in the development cohort. The average of the AUC values obtained on the MIMIC-IV and AUMC development cohorts was used to rank the predictive power of each biomarker. After this, for each patient in the development cohort, we determined the worst recorded value of 40 most predictive physiological parameters during the first day of ICU stay. If a patient had a missing value, we imputed a value, which was considered to lie in the clinically normal range.

We then applied an AUC maximisation procedure to find a statistically optimal, data-driven score for predicting in-hospital mortality within the cohort (technical details described in eAppendix: AUC maximisation). The procedure chose both the optimal features and the four most relevant thresholds for those features. This procedure was applied to select the most predictive biomarker for some of the currently existing organ dysfunction domains of the SOFA score: respiratory, cardiovascular, coagulation, liver, central nervous system, and renal. For the cardiovascular component, we also inspected the predictive performance of the vasopressor-adjusted mean arterial pressure (MAP). The MAP value was adjusted by converting all vasopressors to norepinephrine equivalents^21^ and subtracting the value multiplied by a factor β. More details, with a worked example and the conversion table used to compute norepinephrine equivalents, are given in the eAppendix: vasopressor-adjusted MAP.

For the central nervous system domain, we investigated what was the most predictive way of incorporating information on patient sedation status into the evaluation of the Glasgow Coma Scale (GCS) score. To this end, we compared three options: (i) setting the GCS score to the maximal value of 15 whenever the patient is sedated; (ii) for the duration of the sedation window, using the latest available GCS value prior to the sedation window; and (iii) ignoring the sedation information and using the raw GCS values as recorded in the databases.

Sedation was determined by administration of commonly used medication for sedation: propofol, fentanyl, diazepam, lorazepam, midazolam, oxazepam, methadone, morphine, and hydromorphone. Alternatively, we also investigated how the results differed when we used a sedation criterion based on the Richmond Agitation-Sedation Scale, with sedation defined as a Richmond Agitation-Sedation Scale score ≤−2.

An additional domain—metabolic—was assessed for predictive power in our exploratory analyses. The same AUC maximisation procedure was used to determine the most predictive biomarker in this additional domain.

Since blood gas measurements may be less frequently available in low- and middle-income countries (LMICs), we developed an alternative score called SOFA-LMIC (again using AUC maximisation), in which blood gas markers were replaced with appropriate alternative markers that are more likely to be available in ICUs with fewer resources.

We investigate how removing certain features (and replacing them with alternative ones) affected the score performance in order to understand if multiple scores with near-optimal performance exist. For each organ domain, we determined the most predictive biomarker that was selected in the newly constructed score. Then, for each domain, we removed this biomarker and reconstructed the score with an alternative feature used instead (the exception was the neurological domain, in which the GCS score was the only available feature). This yielded six different scores, each of which used a replacement biomarker instead of the most predictive one (for each of the six domains). We then continued by removing combinations of any two of the most predictive biomarkers, and so on, until all six of the optimal markers were removed. We compared the predictive performance of each score constructed in this way, against the optimal predictive score without any restriction on the choice of biomarkers.

### 2.4 Evaluating the score

The two metrics used for performance evaluation were AUROC and the area under the precision recall curve. These two metrics are explained in the eAppendix: AUC maximisation. Additionally, average mortality rates for each level of our score and the SOFA score, in every organ failure domain, were also assessed.

The score generated by our framework was evaluated at multiple time points in the first day of ICU stay—starting from 6 h into ICU stay up to 24 h into ICU stay—in time steps of 2 h. Patients who were alive and in the ICU at time T = t hours were considered, and for each feature, the worst recorded value over the preceding 24 h was used. As a baseline comparison, the SOFA score was evaluated simultaneously for the same group of patients at the same time point. This analysis was repeated with the metabolic component of our score removed to understand the difference in performance when the two scores were compared across the same organ failure domains. This analysis was further repeated in the cohort of patients who had antibiotics administered on at least 4 consecutive days.

We reported the point estimates and confidence intervals (CIs) obtained by applying bootstrap. The validation was performed on three cohorts—the remaining 40% of the MIMIC-IV (n = 17,402) and AUMC (n = 2169) cohorts, which were not used for score development, and the entire SICdb (n = 2897) cohort. We also assessed AUROCs of all the components of our score at 24 h into ICU stay and compared them to the AUROCs of the SOFA components.

Finally, we assessed the calibration of our score and the SOFA score. For this, we separately fitted logistic regression models on the development cohorts of MIMIC-IV and AUMC to convert the scores to predicted probabilities of death. Then, we investigated the calibration belt of these probabilities when applied to validation cohorts of MIMIC-IV, AUMC, and SICdb separately. We report the slopes and the intercepts of these curves with their respective 95% CIs.

## 3 Results

We evaluated 28,100 (5,339; 2450) suspected septic patients comprising 618,671 (121,673; 58,793) MAP measurements; 63,026 (10,203; 3452) blood urea nitrogen measurements; 56,403 (22,414; 21,950) lactate measurements; 19,736 (11,202; 1990) aspartate aminotransferase measurements; 102,287 (19,056; 37,356) platelet measurements; 193,124 (12,648; 1726) GCS score measurements; and 107,508 (90,174; 31,921) PaO2-to-FiO2 ratios (PaO2 is the partial pressure of oxygen in arterial blood, FiO2 fraction of inspired oxygen). The comprehensive list of all 56 evaluated biomarkers, together with their AUROCs and measurement counts, are given in eTable 1. Information about variable missingness is shown in eTable 2. The comparison of different options for taking into account the sedation status when evaluating the GCS score is shown in eFig. 1.

MIMIC-IV patients had the shortest average length of ICU stay and the lowest SOFA score at 24 h into ICU stay. The average age was 65 years for MIMIC-IV and AUMC, whereas it was 70 years for SICdb. All cohorts had a higher proportion of men. Patient characteristics for each dataset are presented in Table 1.

**Table 1:** Patient characteristics.

Variable	Reported	MIMIC-IV	AUMC	SICdb
Cohort size	n	28 100	5339	2450
Age (years)	Median (IQR)	65 (54–76)	65 (55–75)	70 (60–80)
Admission type				
- Medical	%	65	43	0
- Surgical	%	34	47	100
- Other	%	1	10	0
Mortality	%	10.8	26.0	20.6
ICU LOS	Median (IQR)	2.24 (1.26–4.48)	5.56 (2.54–12.76)	4.27 (2.08–8.83)
Hospital LOS (days)	Median (IQR)	7.79 (4.91–13.25)	NR	NR
Gender (female)	%	43	36	39
Gender (male)	%	57	64	61
SOFA at 24 h	Median (IQR)	5 (3–7)	8 (6–10)	8 (5–10)
- Respiratory	Median (IQR)	1 (0–3)	3 (2–4)	2 (2–3)
- Coagulation	Median (IQR)	0 (0–1)	0 (0–1)	0 (0–1)
- Hepatic	Median (IQR)	0 (0–0)	0 (0–0)	0 (0–0)
- Cardiovascular	Median (IQR)	1 (1–1)	4 (1–4)	3 (1–4)
- CNS	Median (IQR)	0 (0–1)	0 (0–0)	0 (0–1)
- Renal	Median (IQR)	0 (0–1)	0 (0–1)	0 (0–2)

AUMC: Amsterdam University Medical Center; CNS: central nervous system; ICU: intensive care unit; IQR: interquartile range; LOS: length of stay; MIMIC-IV: Medical Information Mart for Intensive Care; NR: not reported; SICdb: Salzburg Intensive Care Database; SOFA: Sequential Organ Failure Assessment.

**Fig. 2:**
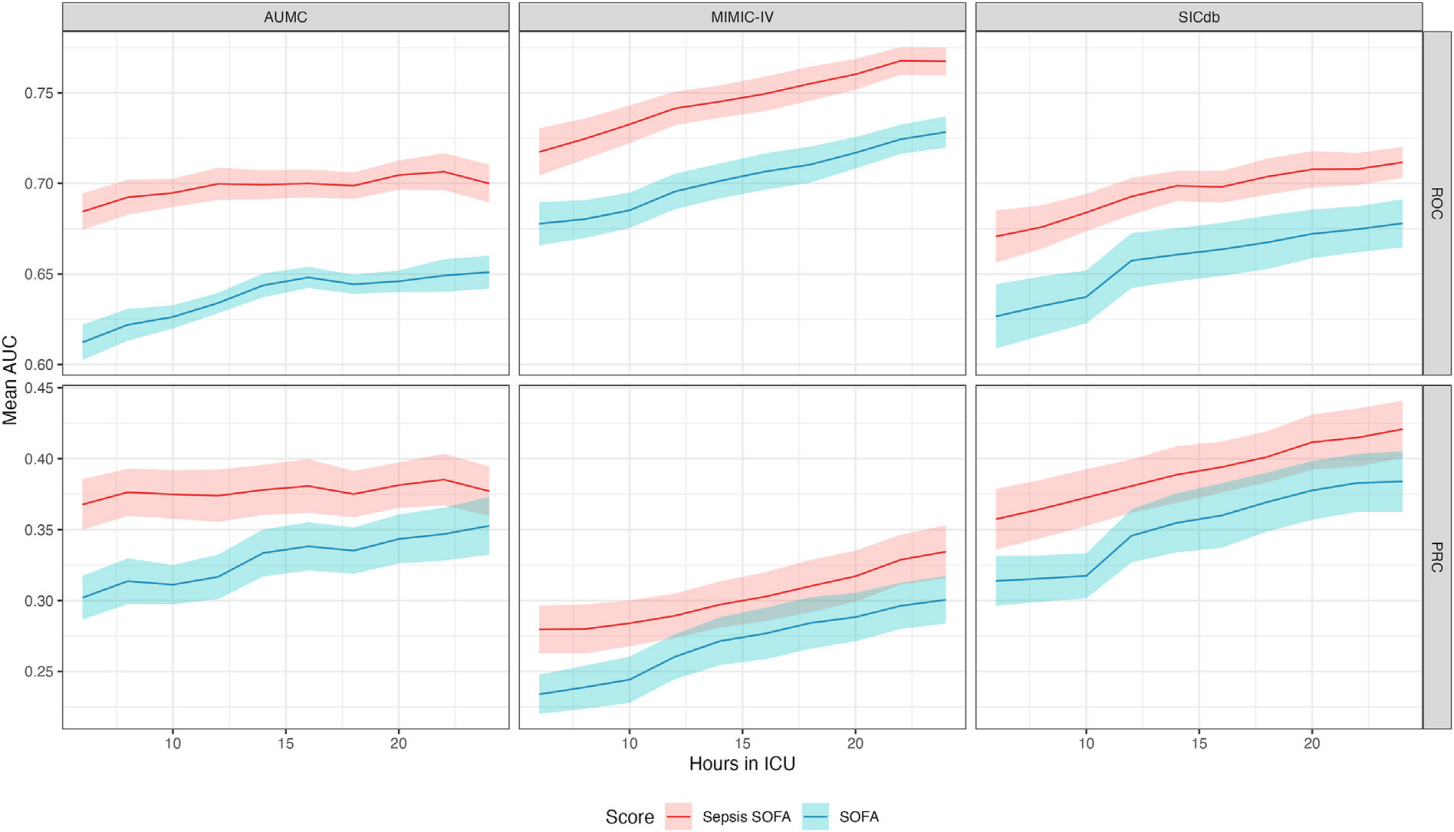
**Over-time performance of SOFA 2.0 and SOFA scores.** SOFA 2.0 and SOFA are evaluated in terms of area under receiver operating characteristic (AUROC) and area under the precision recall curve (AUPRC) of predicting mortality, during the first day of ICU stay, in time steps of 2 h. The 95% confidence intervals for the areas under the curve, obtained using bootstrap, are plotted in every subplot. SOFA 2.0 outperformed SOFA in each metric, time point, and dataset. AUMC: Amsterdam University Medical Center; ICU: intensive care unit; MIMIC-IV: Medical Information Mart for Intensive Care; SICdb: Salzburg Intensive Care Database; SOFA: Sequential Organ Failure Assessment.

The Sepsis SOFA score generated by our framework is presented in Table 2. The comparison of its performance to that of SOFA on the 40% validation MIMIC-IV and AUMC, and entire SICdb cohort is presented in Fig. 2. The alternative score outperformed SOFA significantly in all the three databases and at all times in the first day of ICU stay (at 24 h into ICU AUROC for MIMIC-IV patients: 0.766 vs. 0.727; for AUMC patients: 0.70 vs. 0.653; for SICdb patients: 0.704 vs. 0.665; all p < 0.01). The PRC findings further confirmed these observations (Fig. 1). When removing the metabolic component and when analysing the cohort of patients who received antibiotics for at least 4 days, the score still outperformed SOFA on all three databases (eFigs. 2 and 3, respectively).

**Table 2:** The data-driven Sepsis Sequential Organ Failure Assessment (Sepsis SOFA) score. For low- and middle-income countries (LMICs), replacement features (which do not require readings from a blood gas machine) for the metabolic and respiratory components are provided in parentheses.

Score	Hepatic	Renal	CNS	Metabolic	Cardio	Respiratory	Coagulation
1	Aspartate aminotransferase ≥ 45 IU/L	Blood urea nitrogen ≥ 15 mg/dL	GCS (sedation-adjusted) < 12	Lactate ≥ 1 mmol/L (bicarbonate <19 mEq/L)	Mean arterial pressure—200∗NEQ <80	PaO2-to-FiO2 ratio < 350 mmHg (SpO2-to-FiO2 ratio < 400)	Platelets < 70
2	Aspartate aminotransferase ≥ 50 IU/L	Blood urea nitrogen ≥ 20 mg/dL	GCS (sedation-adjusted) < 8	Lactate ≥ 4 mmol/L (bicarbonate < 17 mEq/L)	Mean arterial pressure—200∗NEQ <40	PaO2-to-FiO2 ratio < 150 mmHg (SpO2-to-FiO2 ratio < 350)	Platelets < 60
3	Aspartate aminotransferase ≥ 195 IU/L	Blood urea nitrogen ≥ 25 mg/dL	GCS (sedation-adjusted) < 7	Lactate ≥ 6 mmol/L (bicarbonate < 16 mEq/L)	Mean arterial pressure—200∗NEQ <20	PaO2-to-FiO2 ratio < 100 mmHg (SpO2-to-FiO2 ratio < 300)	Platelets < 40
4	Aspartate aminotransferase ≥ 225 IU/L	Blood urea nitrogen ≥ 35 mg/dL	GCS (sedation-adjusted) < 6	Lactate ≥ 7 mmol/L (bicarbonate < 10 mEq/L)	Mean arterial pressure—200∗NEQ <5	PaO2-to-FiO2 ratio < 50 mmHg (SpO2-to-FiO2 ratio < 250)	Platelets < 30

∗Sedation-adjusted Glasgow Coma Scale is computed by setting the value to 15 (maximum) for patients receiving sedative drugs including propofol, fentanyl, diazepam, lorazepam, midazolam, oxazepam, methadone, morphine, and hydromorphone.

∗CNS: central nervous system; FiO2: fraction of inspired oxygen; GCS: Glasgow Coma Scale; IU: international unit; MAP: mean arterial pressure; NEQ: norepinephrine equivalent (see eAppendix: vasopressor-adjusted MAP); PaO2: partial pressure of oxygen in arterial blood; SpO2: peripheral oxygen saturation.

The predictive score for LMICs is presented in Table 2, with replacement features (requiring no blood gas machine readings) provided within parentheses. The metabolic component of the score that was based on lactate is replaced with bicarbonate, and the respiratory component based on the PaO2-to-FiO2 ratio was replaced by the SpO2-to-FiO2 ratio. The predictive performance of such a score was again superior to that of the SOFA score (eFig. 4).

The AUROCs of each component of the new predictive score compared with the SOFA score at 24 h into ICU stay are given in eFig. 5 for each dataset. The AUROC and the area under the precision recall curve of each component and dataset are additionally reported in eTable 3. The average mortality rate in each score group of the new score and the SOFA score, for each organ failure domain and each dataset, are presented as bar plots in eFig. 6.

The ablation analysis revealed that even if most predictive features from multiple domains were removed, and alternative features were used to replace them, it was still possible to obtain nearly optimal scores (eFig. 7). This indicated that there may be a multiplicity of scores close to the optimal performance.

The calibration analysis (eFig. 8) revealed that our score, calibrated to MIMIC-IV development cohort, was well calibrated to the MIMIC-IV validation cohort and similarly so for the AUMC database (the intercept CIs contained 0, and the slope CIs contained 1). However, MIMIC-IV development cohort probabilities were not well calibrated to AUMC and SICdb validation cohorts. AUMC development cohort probabilities were not well calibrated to the MIMIC-IV validation cohort, but they were well calibrated to the SICdb validation cohort. In conclusion, calibration of scores worked well internally (between development and validation cohorts) and across European datasets (AUMC and SICdb) but not across continents. Analogous findings were observed for the SOFA score (eFig. 9).

## 4 Discussion

### 4.1 Key findings

We developed a framework that provides an evidence-based, statistically robust, data-driven approach for updating the definition and characterisation of organ failure with enhanced predictive power for mortality in patients with suspected sepsis.

Using data from three large international critical care cohorts to identify the optimally predictive features within each domain of organ dysfunction, we demonstrated improved predictive performance—at different times over the entire first day of ICU stay—when compared with the SOFA score. Furthermore, we identified an additional organ dysfunction domain of significance—metabolic—which consistently displayed strong predictive power. In this way, we provide a methodology for a robust and data-driven way to update the SOFA score, which can be used by consensus groups looking to reform the SOFA score.

### 4.2 Relationship to previous literature

The use of organ failure scores to predict outcome in critical illness is commonplace.[4], [5], [6], [7], [8], [9], [10], [11], [12] We compared the predictive ability of a new, data-driven score versus SOFA in suspected septic patients. We used the term “suspected septic” because sepsis requires the presence of suspected infection and organ dysfunction, with organ dysfunction being the subject matter of our investigation. Thus, the identification of its presence or absence could only occur post hoc. In particular, the consensus definition of sepsis currently necessitates the use of the SOFA score.^2^ Thus, to avoid a cognitive tautology, we had to choose a population that was a candidate for its application (suspected sepsis) rather than the one already defined by the application of SOFA. We acknowledge that the incorporation of SOFA into the Sepsis-3 consensus definition reflected a balance of historical acceptance, pragmatism, and predictive performance in the characterisation of organ dysfunction.^22^^,^^23^ Our study demonstrates, however, that the current acceleration in critical care data acquisition and the evolution of Big Data processing techniques can facilitate a convergence of higher predictive performance and ease of clinical implementation in organ dysfunction scoring systems. These processes can then be applied to the rapid identification and definition of sepsis.^13^

The use of large data sets led to some changes in the variables used to describe some organ dysfunctions. For example, aspartate aminotransferase had greater discriminatory value than bilirubin. Similarly, adjusting mean arterial blood pressure according to the dose of norepinephrine-equivalent vasopressor support also increased the discrimination of the cardiovascular system CVS score. This way of incorporating organ support was designed to be less sensitive to varying clinical practices (such as differences in vasopressor administration between Europe and the United States) than to using thresholds based on vasopressor dosage itself. Finally, for the renal system, urea had better discriminative power for mortality than creatinine. As urea is both a marker of renal function and catabolism, it makes clinical sense for it to carry a greater weight in relation to the prediction of mortality. Our findings also illustrate that there may exist a multiplicity of scores that have nearly optimal performance, and choosing amongst these scores may require further practical considerations, such as how well established a predictive biomarker is amongst practitioners.

The inclusion of lactate as a new metabolic domain is logical: its value as a predictor of outcome in sepsis is well established,^24^^,^^25^ its utilisation as marker for a new “metabolic” dysfunction domain appears physiologically, biologically, and clinically justified, and its availability is now widespread. This was not the case when the SOFA score was first described 28 years ago. In our framework, we assessed the predictive power of adding this new domain and found that it contributed significantly to predictive power. Its noninclusion in the SOFA score may have led and probably still leads to a less comprehensive organ dysfunction function assessment in sepsis. Nonetheless, even with its exclusion, it was possible to construct a data-driven score that outperformed the original SOFA.

In our framework, we focussed on the simplicity and predictive power of a newly developed data-driven score whilst incorporating minimal information about treatment. Other considerations for revising the SOFA score include how well established specific biomarkers are and how their clinical significance is perceived by ICU clinicians. Our framework allows for the incorporation of such specific desiderata and may be used by future consensus groups looking to revise the SOFA score.^13^

### 4.3 Implications of study findings

Our findings imply that it is possible to construct a data-based score of organ dysfunction severity in the Sepsis-3 era. Using such a score, which remains easily implemented at the bedside, allows more accurate and precise prognostication in those patients with suspected sepsis who would be candidates for organ dysfunction assessment. Furthermore, our findings imply that it may become increasingly possible to develop data-based definitions of organ dysfunction with the use of Big Data. This approach may be suitable for iterative updates to the SOFA score in the future. Finally, our findings suggest that a future Sepsis-3 definition with a more data-driven and robust assessment of the presence and severity of organ dysfunction may assist with the stratification of septic patients in randomised controlled trials.

### 4.4 Strengths and limitations

The first and foremost strengths of our study are its size and the heterogeneity of cohorts. We studied information from over 35,000 patients in three varied, international cohorts. The first cohort consists of predominantly medical admissions and was collected in the United States, whilst the second cohort was predominantly made up of surgical admissions and was collected in the Netherlands. The third cohort, collected in Austria, comprised surgical patients. Furthermore, where relevant, we aimed to produce a score that reduced the possible confounding effect of specific hospital treatment practice. Thus, our cardiovascular component accounts for the use, dose, and choice of vasopressors. Finally, our approach was agnostic to variable selection. Thus, we only grouped biomarkers into different organ failure categories and let our statistical model choose the optimal score components and the respective thresholds. Such an approach was used in an attempt to minimise inductive bias.

We acknowledge several limitations. The retrospective design of the study allows for the presence of systematic sampling bias. As a result, we accept that under a different sampling regime, in which the data were collected prospectively and multiple covariates were measured at the same time points, a different predictive score might have emerged. However, this has been a frequent limitation of organ dysfunction assessment since its inception. Moreover, these limitations applied to data-driven and usual SOFA scores equally. However, the data-driven score we derived in this study outperformed the SOFA score in three diverse datasets, all of which reflect how the data are currently collected in practice. Another limitation is that our score uses biomarkers collected using blood gas machines, so the score may be less applicable to under-resourced ICU environments. However, we accounted for this by providing alternative variables for organ assessment that are expected to be available in resource-limited countries. Furthermore, development of scores like ours might guide the decision as to which parameters are important to measure in ICUs with fewer resources. Two of the three datasets used had significant proportions of surgical patients, who may be on prophylactic antibiotic treatment, which may induce a bias in the suspected infection definition. However, we note that our score outperformed SOFA in the cohort of patients who received antibiotics for at least 4 consecutive days. Furthermore, we acknowledge that when adjusting MAP by the amount of administered vasopressors, our data did not include the formulation of norepinephrine (base/tartrate/bitartrate), which may mean there was a discrepancy across datasets. We also acknowledge that the calibration of a data-driven score did not transfer across continents and that the score needs to be calibrated locally. This most likely reflects differences in variable testing, body fluid sampling, and antibiotic administration between Europe and the United States, resulting in different suspected infection cohorts. This was reflected in a higher prevalence of suspected infection, and reduced mortality, in the US cohort than in the cohort from Europe. However, the same issue was present for the original SOFA score, and our analysis highlights this previously unappreciated issue. Our updated SOFA score was developed in a unique population of patients with suspected sepsis. Its predictive performance in the wider population of critically ill patients without sepsis remains untested. Thus, its findings cannot be used to derive a global novel SOFA score to be applied to all critically ill patients. Finally, even though our study includes three large, heterogeneous datasets, the external validity of our derived organ dysfunction score remains a paramount consideration. In the future, we hope to evaluate our score on more datasets, both retrospectively and prospectively.

## 5 Conclusion

In an international, multicohort ICU study, we developed a framework for deriving a data-based update to the SOFA score, which can be used to develop a score for the identification and classification of organ dysfunction in patients with suspected sepsis. Such a data-driven score outperformed the SOFA score in all the databases we investigated. Thus, it represents a novel and superior approach to the identification and classification of organ dysfunction in patients with suspected sepsis. Further studies by expert consensus groups are required to determine exactly which variables are sufficiently pragmatic and clinically acceptable to be used in conjunction with a data-driven update to the SOFA score. In this regard, the framework developed in this paper is suitable for incorporating further clinical desiderata based on expert opinion.

## Consent to participate

Not applicable.

## CRediT authorship contribution statement

Drago Plečko and Nicolas Bennett developed the study design. Drago Plečko designed and conducted the statistical analysis. Drago Plečko, Ida-Fong Ukor, and Ary Serpa-Neto wrote the manuscript. Drago Plečko and Nicolas Bennett developed the supporting computational infrastructure. Nicolas Bennett, Ida-Fong Ukor, Peter Bühlmann, Niklas Rodemund, and Ary Serpa-Neto revised the manuscript.

## Availability of data and material

All the data used are publicly available.

## Code availability

All code is available on https://github.com/eth-mds/dose.

## Consent for publication

All authors consent to publication of the manuscript.

## Funding

Authors Drago Plečko and Nicolas Bennett are supported by the grant #2017-110 of the Strategic Focal Area “Personalized Health and Related Technologies (PHRT)” of the ETH (Eidgenössische Technische Hochschule) Zuricho Domain for the Swiss Personalized Health Network/PHRT Driver Project “Personalized Swiss Sepsis Study”. Other authors are funded by their respective institutions.

## Conflict of interest

The authors declare that they have no known competing financial interests or personal relationships that could have appeared to influence the work reported in this paper.
