# Supplementary material for: A framework and analytical exploration for a data-driven update of the Sequential Organ Failure Assessment (SOFA) score in sepsis
Source: Crit Care Resusc. 2025 Mar 14;27(1):100105. doi: 10.1016/j.ccrj.2025.100105 (PMC11952785; doi:10.1016/j.ccrj.2025.100105)
Supplement: Multimedia component 1 [file mmc1.docx]

eTable 1. Comprehensive list of features that were assessed for predictive power in predicting mortality within the suspected infection cohorts. Area under receiver operator characteristic (AUC) for the MIMIC-IV and AUMC cohorts (development cohorts) is reported. Number of measurements of each feature within the first 24 hours, median and IQR values are presented for each database. The organ failure category of each biomarker is also included.

| Variable (unit) | AUROC | | n (n/pp) | | | Median [IQR] | | | Category |
| --- | --- | --- | --- | --- | --- | --- | --- | --- | --- |
|  | MIMIC-IV | AUMC | MIMIC-IV | AUMC | SICdb | MIMIC-IV | AUMC | SICdb |  |
| Aspartate Aminotransferase (IU/L) | 0.608 | 0.621 | 19736 (1) | 11202 (2) | 1990 (1) | 61 [29, 192] | 84 [37, 240] | 49 [26, 118] | Hepatic |
| Alanine Aminotransferase (IU/L) | 0.557 | 0.603 | 19678 (1) | 11304 (2) | 2029 (1) | 41 [20, 132] | 52 [24, 141] | 32 [18, 75] |  |
| Albumin (g/dL) | 0.573 | 0.55 | 10819 (0) | 8070 (2) | 0 (0) | 3 [2.5, 3.4] | 2.4 [1.8, 2.9] | N/A |  |
| Alkaline Phosphatase (IU/L) | 0.545 | 0.571 | 19362 (1) | 8738 (2) | 2001 (1) | 84 [59, 130] | 70 [51, 100] | 80 [53, 128] |  |
| Total bilirubin (mg/dL) | 0.536 | 0.518 | 19709 (1) | 8254 (2) | 2004 (1) | 0.9 [0.5, 2.3] | 0.6 [0.4, 0.9] | 0.8 [0.5, 1.6] |  |
| Direct Bilirubin (mg/dL) | 0.518 | 0.497 | 2035 (0) | 48 (0) | 444 (0) | 1.8 [0.8, 4] | 0 [0, 0] | 1.8 [1.2, 3.4] |  |
| Blood Urea Nitrogen (mg/dL) | 0.693 | 0.644 | 63026 (3) | 10203 (2) | 4162 (2) | 20 [13, 34] | 21.3 [14.6, 33.3] | 21.9 [14.5, 35] | Renal |
| Creatinine (mg/dL) | 0.655 | 0.629 | 63192 (3) | 13654 (3) | 4071 (2) | 1 [0.7, 1.6] | 1.1 [0.8, 1.6] | 1.1 [0.8, 1.7] |  |
| Calcium (mg/dL) | 0.605 | 0.56 | 51840 (2) | 14374 (3) | 4112 (2) | 8.2 [7.7, 8.7] | 7.8 [7.3, 8.3] | 8.5 [8.1, 8.8] |  |
| Magnesium (mg/dL) | 0.565 | 0.595 | 56294 (2) | 15559 (3) | 3962 (2) | 2 [1.8, 2.3] | 2.1 [1.8, 2.5] | 2.1 [1.9, 2.5] |  |
| Potassium (mEq/L) | 0.571 | 0.584 | 66853 (3) | 50973 (11) | 22287 (10) | 4.2 [3.8, 4.6] | 4.1 [3.7, 4.5] | 4.1 [3.8, 4.5] |  |
| Ionized Calcium (mmol/L) | 0.544 | 0.565 | 47889 (2) | 38758 (8) | 21970 (10) | 1.1 [1.1, 1.2] | 1.1 [1.1, 1.2] | 1.1 [1.1, 1.2] |  |
| Chloride (mEq/L) | 0.574 | 0.531 | 66405 (3) | 15216 (3) | 22098 (10) | 105 [101, 109] | 109 [105, 113] | 104 [102, 107] |  |
| Sodium (mEq/L) | 0.516 | 0.497 | 65371 (3) | 50526 (10) | 20782 (10) | 138 [136, 141] | 139 [136, 142] | 138.2 [135.4, 141] |  |
| GCS (points) | 0.586 | 0.544 | 193124 (8) | 12648 (3) | 1726 (1) | 14 [8, 15] | 11 [7, 14] | 14 [11, 15] | CNS |
| GCS (sedation adjusted) (points) | 0.536 | 0.527 | 193124 (8) | 12648 (3) | 1726 (1) | 15 [15, 15] | 15 [14, 15] | 14 [11, 15] |  |
| Lactate (mmol/L) | 0.636 | 0.653 | 56403 (2) | 22414 (5) | 21950 (10) | 2.1 [1.4, 3.4] | 2.1 [1.2, 3.9] | 1.6 [1.1, 2.6] | Metabolic |
| pH of Blood (-) | 0.63 | 0.627 | 87796 (4) | 48996 (10) | 21766 (10) | 7.4 [7.3, 7.4] | 7.3 [7.3, 7.4] | 7.4 [7.3, 7.4] |  |
| Bicarbonate (mEq/L) | 0.644 | 0.611 | 62877 (3) | 48838 (10) | 21903 (10) | 23 [20, 25] | 21.5 [18.6, 24.1] | 23.7 [20.9, 26.5] |  |
| MAP (mmHg) — 200*NEQ (mcg/kg/min) | 0.656 | 0.603 | 618671 (26) | 121673 (25) | 58793 (27) | 71 [61, 82] | 62 [39.6, 77] | 66.4 [52.2, 78.3] | Cardio |
| MAP (mmHg) — 100*NEQ (mcg/kg/min) | 0.648 | 0.589 | 618671 (26) | 121673 (25) | 58793 (27) | 72 [63, 83] | 66 [52.6, 79] | 68.2 [58.2, 79] |  |
| MAP (mmHg) — 50*NEQ (mcg/kg/min) | 0.635 | 0.572 | 618671 (26) | 121673 (25) | 58793 (27) | 73 [64, 83] | 69.1 [58.5, 81] | 69.6 [61.2, 79.6] |  |
| Systolic BP (mmHg) | 0.605 | 0.545 | 604020 (25) | 120426 (25) | 58512 (27) | 113 [100, 127] | 110 [94, 128] | 107.2 [96.1, 122] |  |
| MAP (mmHg) | 0.603 | 0.52 | 616011 (26) | 120423 (25) | 58702 (27) | 74 [66, 84] | 74 [65, 85] | 71.7 [65.1, 80.6] |  |
| Heart Rate (bpm) | 0.594 | 0.517 | 629476 (26) | 123793 (26) | 58950 (28) | 85 [74, 98] | 87 [70, 104] | 84.4 [72.6, 98] |  |
| Diastolic BP (mmHg) | 0.582 | 0.526 | 603916 (25) | 120377 (25) | 58513 (27) | 59 [51, 68] | 56 [49, 64] | 54.2 [48.2, 61.3] |  |
| Creatine Kinase (IU/L) | 0.528 | 0.519 | 13772 (1) | 14933 (3) | 1213 (1) | 229 [81, 886] | 430 [162, 1369] | 319 [102, 860] |  |
| PaO2/FiO2 (mmHg) | 0.631 | 0.602 | 107508 (4) | 90174 (19) | 31921 (15) | 290 [182.5, 477.5] | 244.4 [167.9, 350] | 264.8 [173.6, 371.4] | Respiratory |
| PaO2 (mmHg) | 0.634 | 0.574 | 78152 (3) | 47835 (10) | 21511 (10) | 124 [87, 206] | 97 [78, 127] | 86.6 [73.9, 105.3] |  |
| SpO2/FiO2 (%) | 0.582 | 0.594 | 623465 (26) | 122402 (25) | 59214 (28) | 442.9 [232.5, 461.9] | 243.9 [195.9, 326.7] | 438.8 [227.8, 458.3] |  |
| PaCO2 (mmHg) | 0.575 | 0.585 | 82064 (3) | 49149 (10) | 21864 (10) | 40 [35, 45] | 39 [34, 45] | 39 [34.4, 44.5] |  |
| FiO2 (%) | 0.562 | 0.563 | 108829 (5) | 94053 (20) | 24907 (12) | 50 [40, 60] | 41 [40, 59] | 45 [35.2, 55.4] |  |
| O2 Saturation (%) | 0.557 | 0.524 | 623870 (26) | 123901 (26) | 59293 (28) | 97 [94, 99] | 97 [94, 99] | 96 [94, 97.6] |  |
| Respiratory Rate (insp/min) | 0.614 | 0.465 | 627677 (26) | 118416 (25) | 22959 (11) | 19 [16, 23] | 21 [16, 27] | 15 [14, 17.9] |  |
| Platelets to INR(PT) Ratio (K/uL/INR) | 0.58 | 0.563 | 66850 (3) | 17883 (4) | 4351 (2) | 132.7 [83.8, 195.8] | 152.1 [94, 218] | 152.1 [89.9, 224.7] | Coagulation |
| APTT (sec) * INR(PT) | 0.623 | 0.499 | 47940 (2) | 6820 (1) | 4361 (2) | 42.8 [33.8, 64] | 58.9 [43.7, 100.8] | 52.1 [38.9, 77.3] |  |
| INR(PT) | 0.614 | 0.507 | 48337 (2) | 6844 (1) | 4254 (2) | 1.3 [1.2, 1.6] | 1.3 [1.2, 1.7] | 1.4 [1.2, 1.7] |  |
| Platelets (K/uL) | 0.536 | 0.553 | 64858 (3) | 16541 (3) | 4262 (2) | 165 [115, 230] | 177 [116, 244] | 206 [145, 287] |  |
| Phosphate (mg/dL) | 0.654 | 0.63 | 52115 (2) | 14878 (3) | 3970 (2) | 3.5 [2.8, 4.4] | 3.4 [2.6, 4.4] | 3.9 [3.2, 4.7] | Other |
| Base Excess (mEq/L) | 0.633 | 0.627 | 81981 (3) | 48677 (10) | 21849 (10) | -1 [-4, 0] | -3.6 [-7.1, -0.5] | -1 [-4, 1.9] |  |
| Partial Thromboplastin Time (sec) | 0.6 | 0.564 | 49327 (2) | 6847 (1) | 4481 (2) | 32 [27.9, 40.6] | 42 [36, 57] | 38 [32, 47] |  |
| Glucose (mg/dL) | 0.554 | 0.608 | 95603 (4) | 51631 (11) | 22376 (10) | 132 [109, 164] | 142.3 [117.1, 176.6] | 125 [102, 153] |  |
| RDW (%) | 0.658 | 0.501 | 63283 (3) | 8 (0) | 0 (0) | 14.5 [13.5, 16] | 13.9 [13.1, 15.1] | N/A |  |
| Troponin T (ng/mL) | 0.549 | 0.584 | 11815 (0) | 6161 (1) | 1065 (0) | 0.1 [0, 0.6] | 0.3 [0.1, 1.2] | 0.2 [0, 0.8] |  |
| MCHC (%) | 0.62 | 0.5 | 63323 (3) | 553 (0) | 4269 (2) | 33.3 [32.2, 34.4] | 33.8 [33.8, 35.4] | 33.8 [32.8, 34.7] |  |
| Lymphocytes (%) | 0.601 | 0.515 | 13810 (1) | 2587 (1) | 111 (0) | 9.7 [5.3, 16] | 7.8 [5, 12] | 7.8 [4.9, 11.3] |  |
| Basophils (%) | 0.578 | 0.527 | 13810 (1) | 2572 (1) | 109 (0) | 0.2 [0, 0.3] | 0 [0, 0.2] | 0.2 [0.1, 0.4] |  |
| Prothrombine Time (sec) | 0.606 | 0.498 | 48347 (2) | 281 (0) | 549 (0) | 14.5 [12.9, 17.2] | 1.4 [1.2, 1.8] | 19 [17, 22] |  |
| MCV (fL) | 0.592 | 0.51 | 63301 (3) | 2051 (0) | 4269 (2) | 91 [87, 95] | 90 [87, 94] | 88.6 [84.8, 92.4] |  |
| WBC (K/uL) | 0.565 | 0.524 | 63406 (3) | 14523 (3) | 4261 (2) | 11.7 [8.3, 16.1] | 12.3 [8.4, 17.2] | 11.9 [8.1, 16.9] |  |
| Eosinophils (%) | 0.597 | 0.483 | 13808 (1) | 1609 (0) | 109 (0) | 0.4 [0, 1.1] | 0.4 [0.2, 0.8] | 0.1 [0, 0.6] |  |
| Hemoglobin (g/dL) | 0.54 | 0.515 | 64398 (3) | 48036 (10) | 23055 (11) | 10.2 [8.9, 11.6] | 11.1 [9.7, 12.9] | 10.2 [8.8, 11.9] |  |
| RBC (m/uL) | 0.538 | 0.517 | 63302 (3) | 2732 (1) | 4267 (2) | 3.4 [3, 3.9] | 3.6 [3.2, 4.2] | 3.4 [2.9, 3.9] |  |
| neutrophils (%) | 0.554 | 0.485 | 13810 (1) | 1634 (0) | 111 (0) | 81.4 [73.5, 87.5] | 84.6 [79.3, 88.1] | 86.5 [78.7, 90] |  |
| Fibrinogen (mg/dL) | 0.541 | 0.494 | 12735 (1) | 619 (0) | 4161 (2) | 212 [162, 305.5] | 230 [170, 330] | 403 [303, 530] |  |
| Creatine Kinase MB (IU/L) | 0.531 | 0.503 | 13695 (1) | 138 (0) | 98 (0) | 7 [3, 19] | 21.6 [7.5, 51.5] | 4.4 [2.1, 20.6] |  |
| Hematocrit (%) | 0.51 | 0.514 | 75607 (3) | 45729 (9) | 22582 (11) | 30.3 [26.7, 34.5] | 34 [29, 39] | 30 [26, 35] |  |
| MCH (pg) | 0.515 | 0.505 | 63289 (3) | 2050 (0) | 4269 (2) | 30.3 [28.9, 31.7] | 30.9 [29.6, 32.2] | 30 [28.7, 31.3] |  |
| Temperature (C) | 0.477 | 0.428 | 212529 (9) | 27297 (6) | 49605 (23) | 36.9 [36.6, 37.3] | 36 [33.1, 36.9] | 36.9 [36.3, 37.4] |  |
| APTT activate plasma thromboplastin time; BP blood pressure; CNS Central Nervous System; GCS Glasgow Coma Scale; FiO2 fraction of inspired oxygen; INR international normalized ratio of prothrombine time; MCH mean corpuscular hemoglobin; MCHC mean corpuscular hemoglobin concentration; MCV mean corpuscular volume; NEQ norepinephrine equivalents (see eAppendix: Vasopressor Adjusted MAP); PaCO2 partial arterial CO2 pressure; PaO2 partial arterial O2 pressure; RBC red blood cells; RDW red cell distribution width; WBC white blood cells. | | | | | | | | | |
