# Supplementary material for: A framework and analytical exploration for a data-driven update of the Sequential Organ Failure Assessment (SOFA) score in sepsis
Source: Crit Care Resusc. 2025 Mar 14;27(1):100105. doi: 10.1016/j.ccrj.2025.100105 (PMC11952785; doi:10.1016/j.ccrj.2025.100105)
Supplement: Multimedia component 2 [file mmc2.docx]

eTable 2. Comprehensive list of features that were assessed for predictive power in predicting mortality within the suspected infection cohorts. The proportion and number of patients with missing values for each feature in the first 24 hours is reported, in the overall cohort for the respective dataset.

| Variable | Missingness on first day (% (n)) | | | Category |
| --- | --- | --- | --- | --- |
|  | MIMIC-IV | AUMC | SICdb |  |
| Alanine Aminotransferase | 53.4% (12893) | 11.6% (558) | 36.3% (778) | Hepatic |
| Aspartate Aminotransferase | 53.2% (12852) | 12.4% (596) | 37.3% (799) |  |
| Alkaline Phosphatase | 53.8% (12994) | 14.7% (711) | 36.8% (789) |  |
| Total bilirubin | 53.6% (12932) | 17.2% (829) | 37.0% (792) |  |
| Albumin | 69.4% (16752) | 12.6% (609) | N/A |  |
| Direct Bilirubin | 95.1% (22971) | 99.2% (4784) | 85.7% (1836) |  |
| Potassium | 0.6% (154) | 0.2% (9) | 0.3% (7) | Renal |
| Sodium | 0.6% (146) | 0.2% (11) | 0.3% (7) |  |
| Creatinine | 0.6% (140) | 1.0% (47) | 3.7% (80) |  |
| Blood Urea Nitrogen | 0.6% (141) | 7.4% (355) | 1.9% (41) |  |
| Magnesium | 5.3% (1274) | 1.8% (88) | 6.3% (134) |  |
| Calcium | 12.3% (2967) | 3.7% (178) | 2.1% (44) |  |
| Chloride | 0.6% (152) | 32.9% (1586) | 0.5% (10) |  |
| Ionized Calcium | 45.5% (10995) | 19.9% (958) | 0.4% (8) |  |
| GCS (sedation adjusted) | 0.2% (54) | 47.0% (2265) | 30.5% (654) | CNS |
| GCS | 0.2% (54) | 47.0% (2265) | 30.5% (654) |  |
| Bicarbonate | 0.5% (132) | 0.7% (34) | 0.5% (10) | Metabolic |
| pH of Blood | 25.3% (6114) | 0.7% (32) | 0.9% (19) |  |
| Lactate | 27.4% (6623) | 11.0% (531) | 0.4% (9) |  |
| Heart Rate | 0.2% (44) | 0.0% (0) | 0.0% (0) | Cardio |
| MAP | 0.2% (45) | 0.8% (39) | 0.0% (0) |  |
| MAP - 50*NEQ | 0.2% (45) | 0.8% (39) | 0.0% (0) |  |
| MAP - 100*NEQ | 0.2% (45) | 0.8% (39) | 0.0% (0) |  |
| MAP (mmHg) — 200*NEQ (mcg/kg/min) | 0.2% (45) | 0.8% (39) | 0.0% (0) |  |
| Systolic BP | 0.5% (113) | 0.8% (38) | 0.0% (0) |  |
| Diastolic BP | 0.5% (114) | 0.8% (39) | 0.0% (0) |  |
| Creatine Kinase | 71.6% (17283) | 5.9% (285) | 70.1% (1501) |  |
| O2 Saturation | 0.1% (26) | 0.0% (1) | 0.0% (0) | Respiratory |
| SpO2/FiO2 | 0.2% (46) | 0.1% (4) | 0.0% (0) |  |
| PaCO2 | 27.3% (6581) | 0.7% (32) | 0.7% (15) |  |
| Respiratory Rate | 0.2% (52) | 0.6% (30) | 28.1% (602) |  |
| PaO2/FiO2 | 29.8% (7205) | 0.7% (35) | 1.3% (28) |  |
| PaO2 | 30.3% (7307) | 0.7% (36) | 1.3% (28) |  |
| FiO2 | 33.4% (8053) | 13.1% (633) | 23.9% (511) |  |
| Platelets to INR(PT) Ratio | 0.7% (181) | 0.8% (38) | 1.9% (41) | Coagulation |
| Platelets | 0.8% (184) | 0.9% (43) | 1.9% (41) |  |
| INR(PT) | 12.0% (2889) | 54.9% (2647) | 3.8% (81) |  |
| APTT (sec) * INR(PT) | 12.9% (3108) | 55.3% (2667) | 4.1% (88) |  |
| Glucose | 0.5% (124) | 0.1% (7) | 0.3% (7) | Other |
| Hemoglobin | 0.8% (190) | 0.3% (13) | 0.4% (8) |  |
| Hematocrit | 0.6% (155) | 0.4% (17) | 0.5% (11) |  |
| WBC | 0.7% (179) | 0.8% (39) | 1.9% (41) |  |
| Phosphate | 11.9% (2878) | 2.3% (110) | 6.4% (138) |  |
| Base Excess | 27.3% (6580) | 0.7% (32) | 0.6% (13) |  |
| RBC | 0.8% (182) | 57.0% (2749) | 1.9% (40) |  |
| MCV | 0.8% (182) | 64.7% (3119) | 1.9% (40) |  |
| MCH | 0.8% (185) | 64.7% (3120) | 1.9% (40) |  |
| Partial Thromboplastin Time | 12.6% (3037) | 55.3% (2665) | 4.1% (88) |  |
| Temperature | 4.8% (1167) | 67.5% (3255) | 2.1% (46) |  |
| MCHC | 0.8% (185) | 90.6% (4369) | 1.9% (40) |  |
| Fibrinogen | 68.2% (16459) | 92.2% (4446) | 6.3% (136) |  |
| Prothrombine Time | 12.0% (2889) | 97.6% (4707) | 83.4% (1787) |  |
| Troponin T | 78.5% (18954) | 48.0% (2314) | 73.8% (1581) |  |
| RDW | 0.8% (189) | 99.9% (4816) | N/A |  |
| Lymphocytes | 56.4% (13630) | 54.8% (2641) | 95.9% (2054) |  |
| Basophils | 56.4% (13630) | 55.0% (2653) | 95.9% (2055) |  |
| neutrophils | 56.4% (13630) | 71.1% (3428) | 95.9% (2054) |  |
| Eosinophils | 56.5% (13632) | 71.4% (3445) | 95.9% (2055) |  |
| Creatine Kinase MB | 73.2% (17674) | 98.8% (4765) | 96.2% (2061) |  |
| APTT activated plasma thromboplastin time; BP blood pressure; CNS Central Nervous System; GCS Glasgow Coma Scale; FiO2 fraction of inspired oxygen; INR international normalized ratio of prothrombine time; MCH mean corpuscular hemoglobin; MCHC mean corpuscular hemoglobin concentration; MCV mean corpuscular volume; NEQ norepinephrine equivalents (see eAppendix: Vasopressor Adjusted MAP); PaCO2 partial arterial CO2 pressure; PaO2 partial arterial O2 pressure; RBC red blood cells; RDW red cell distribution width; WBC white blood cells. | | | | |
