# Supplementary material for: A framework and analytical exploration for a data-driven update of the Sequential Organ Failure Assessment (SOFA) score in sepsis
Source: Crit Care Resusc. 2025 Mar 14;27(1):100105. doi: 10.1016/j.ccrj.2025.100105 (PMC11952785; doi:10.1016/j.ccrj.2025.100105)
Supplement: Multimedia component 3 [file mmc3.docx]

eTable 3A. Area under receiver operator characteristic (AUROC) per component and dataset, for validation and development cohorts.

|  | MIMIC-IV | | | | AUMC | | | | SICdb | |
| --- | --- | --- | --- | --- | --- | --- | --- | --- | --- | --- |
|  | Development | | Validation | | Development | | Validation | | | |
| Component | Sepsis SOFA | SOFA | Sepsis SOFA | SOFA | Sepsis SOFA | SOFA | Sepsis SOFA | SOFA | Sepsis SOFA | SOFA |
| Cardio | 0.64 (0.63-0.66) | 0.64 (0.63-0.65) | 0.64 (0.63-0.66) | 0.64 (0.62-0.66) | 0.59 (0.57-0.61) | 0.57 (0.55-0.59) | 0.60 (0.57-0.63) | 0.57 (0.55-0.60) | 0.62 (0.59-0.64) | 0.59 (0.56-0.61) |
| Hepatic | 0.59 (0.58-0.61) | 0.51 (0.50-0.53) | 0.61 (0.59-0.62) | 0.53 (0.51-0.55) | 0.57 (0.55-0.60) | 0.50 (0.48-0.52) | 0.56 (0.53-0.58) | 0.52 (0.49-0.55) | 0.52 (0.50-0.55) | 0.52 (0.49-0.56) |
| CNS | 0.54 (0.53-0.55) | 0.53 (0.52-0.55) | 0.54 (0.53-0.55) | 0.53 (0.51-0.55) | 0.51 (0.50-0.53) | 0.52 (0.50-0.55) | 0.51 (0.49-0.52) | 0.53 (0.50-0.56) | 0.52 (0.50-0.54) | 0.54 (0.51-0.57) |
| Coagulation | 0.56 (0.55-0.57) | 0.55 (0.54-0.57) | 0.55 (0.54-0.56) | 0.54 (0.53-0.56) | 0.54 (0.53-0.56) | 0.54 (0.52-0.56) | 0.52 (0.51-0.54) | 0.56 (0.53-0.59) | 0.53 (0.52-0.54) | 0.58 (0.55-0.60) |
| Renal | 0.68 (0.67-0.70) | 0.67 (0.65-0.68) | 0.68 (0.67-0.70) | 0.68 (0.66-0.69) | 0.65 (0.62-0.67) | 0.62 (0.60-0.65) | 0.63 (0.61-0.66) | 0.63 (0.60-0.66) | 0.64 (0.61-0.66) | 0.63 (0.60-0.66) |
| Respiratory | 0.56 (0.55-0.57) | 0.61 (0.60-0.63) | 0.57 (0.55-0.58) | 0.62 (0.60-0.63) | 0.56 (0.54-0.58) | 0.58 (0.56-0.61) | 0.57 (0.54-0.59) | 0.57 (0.54-0.60) | 0.54 (0.51-0.56) | 0.59 (0.57-0.62) |
| Metabolic | 0.63 (0.62-0.65) | - | 0.63 (0.61-0.65) | - | 0.65 (0.63-0.67) | - | 0.63 (0.60-0.66) | - | 0.63 (0.60-0.65) | - |

eTable 3B. Area under precision recall (AUPRC) per component and dataset, for validation and development cohorts.

|  | MIMIC-IV | | | | AUMC | | | | HiRID | |
| --- | --- | --- | --- | --- | --- | --- | --- | --- | --- | --- |
|  | Development | | Validation | | Development | | Validation | | | |
| Component | Sepsis SOFA | SOFA | Sepsis SOFA | SOFA | Sepsis SOFA | SOFA | Sepsis SOFA | SOFA | Sepsis SOFA | SOFA |
| Cardio | 0.21 (0.19-0.22) | 0.19 (0.18-0.20) | 0.20 (0.18-0.22) | 0.19 (0.18-0.21) | 0.31 (0.28-0.33) | 0.28 (0.26-0.30) | 0.29 (0.26-0.32) | 0.25 (0.23-0.28) | 0.31 (0.28-0.35) | 0.26 (0.24-0.29) |
| Hepatic | 0.16 (0.15-0.17) | 0.15 (0.14-0.17) | 0.18 (0.16-0.20) | 0.17 (0.15-0.19) | 0.31 (0.28-0.34) | 0.28 (0.25-0.31) | 0.26 (0.23-0.30) | 0.26 (0.23-0.30) | 0.23 (0.21-0.26) | 0.26 (0.23-0.30) |
| CNS | 0.13 (0.12-0.14) | 0.13 (0.12-0.14) | 0.13 (0.12-0.14) | 0.13 (0.12-0.14) | 0.27 (0.24-0.29) | 0.27 (0.24-0.29) | 0.23 (0.21-0.26) | 0.24 (0.21-0.26) | 0.24 (0.21-0.27) | 0.25 (0.22-0.27) |
| Coagulation | 0.16 (0.14-0.17) | 0.15 (0.14-0.17) | 0.15 (0.14-0.17) | 0.15 (0.13-0.17) | 0.30 (0.28-0.33) | 0.30 (0.27-0.32) | 0.25 (0.22-0.28) | 0.26 (0.23-0.30) | 0.26 (0.23-0.29) | 0.29 (0.26-0.32) |
| Renal | 0.18 (0.17-0.19) | 0.20 (0.18-0.21) | 0.19 (0.17-0.20) | 0.20 (0.19-0.22) | 0.34 (0.31-0.37) | 0.35 (0.32-0.39) | 0.30 (0.27-0.34) | 0.33 (0.29-0.37) | 0.30 (0.27-0.33) | 0.33 (0.29-0.36) |
| Respiratory | 0.15 (0.14-0.16) | 0.17 (0.16-0.18) | 0.16 (0.14-0.18) | 0.17 (0.15-0.18) | 0.29 (0.27-0.32) | 0.29 (0.27-0.31) | 0.28 (0.25-0.32) | 0.26 (0.23-0.28) | 0.26 (0.22-0.29) | 0.28 (0.25-0.31) |
| Metabolic | 0.21 (0.19-0.22) | - | 0.21 (0.19-0.23) | - | 0.38 (0.35-0.41) | - | 0.33 (0.29-0.37) | - | 0.35 (0.31-0.38) | - |
