# Supplementary material for: A framework and analytical exploration for a data-driven update of the Sequential Organ Failure Assessment (SOFA) score in sepsis
Source: Crit Care Resusc. 2025 Mar 14;27(1):100105. doi: 10.1016/j.ccrj.2025.100105 (PMC11952785; doi:10.1016/j.ccrj.2025.100105)
Supplement: Multimedia component 4 [file mmc4.pdf]

A FRAMEWORK AND ANALYTICAL EXPLORATION FOR A DATA-DRIVEN UPDATE OF THE SEQUENTIAL ORGAN FAILURE ASSESSMENT (SOFA) SCORE IN SEPSIS
eFigures

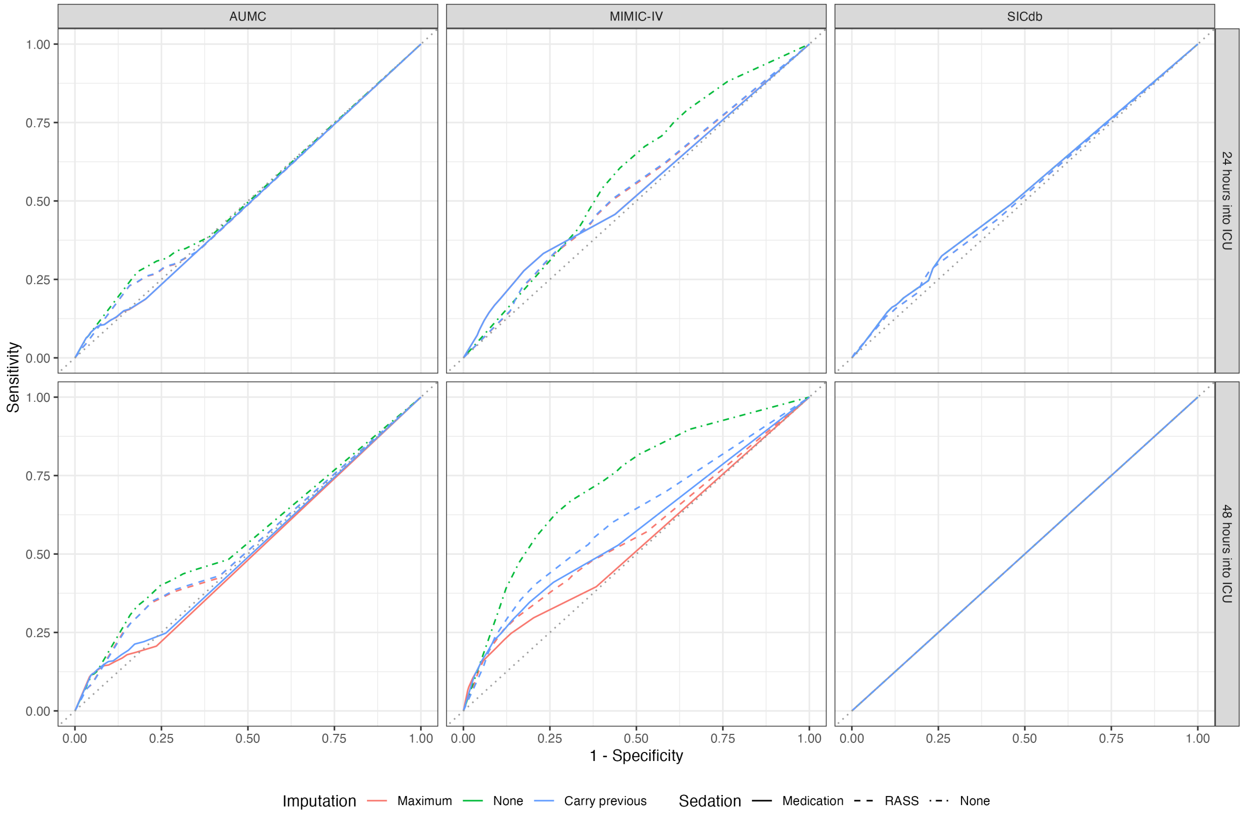

eFigure 1: Comparison of sedation and imputation options for the central nervous system component. In a post-hoc analysis, we inspected the predictive performance of three imputation techniques: (i) setting the GCS score to the maximal value whenever the patient is sedated; (ii) for the duration of the sedation window, using the latest available GCS value prior to the sedation window; (iii) ignoring the sedation information and using the raw GCS values as recorded in the databases, and two approaches to sedation: (a) medication based; (b) RASS based; (c) ignoring sedation. Our findings show that raw GCS values with sedation status not taken into account achieve the highest marginal AUC for predicting mortality. However, sedation-adjusted values (setting the GCS score to 15 based on administration of sedative drugs) are used by the data-driven predictive Sepsis SOFA score.

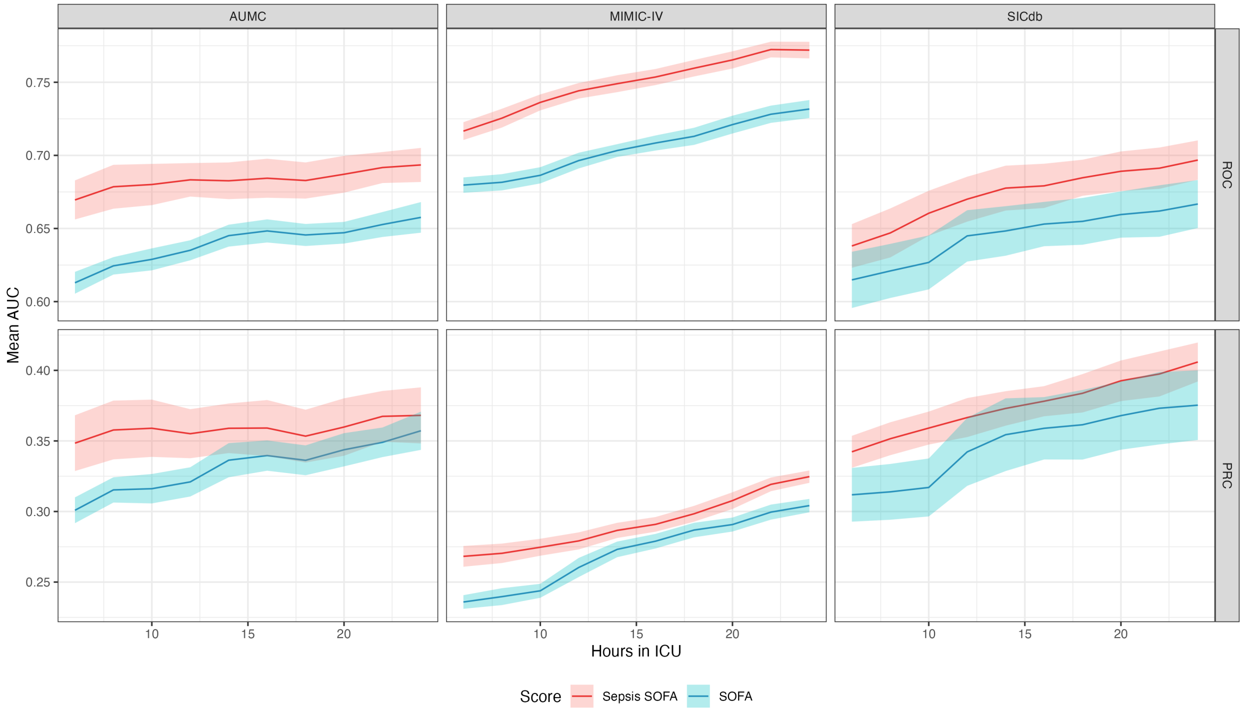

eFigure 2: Over-time performance of the data-driven Sepsis SOFA and SOFA scores, with the metabolic component of the new score removed. The two scores are evaluated in terms of area under receiver operator characteristic (AUROC) and area under precision recall (AUPRC) for predicting mortality, during the first day of ICU stay, in time steps of 2 hours, with the metabolic component of Sepsis SOFA not included. The 95% confidence intervals for the areas under the curve, obtained using bootstrap, are plotted in every subplot. The new score still outperforms SOFA in each metric, time point and dataset.

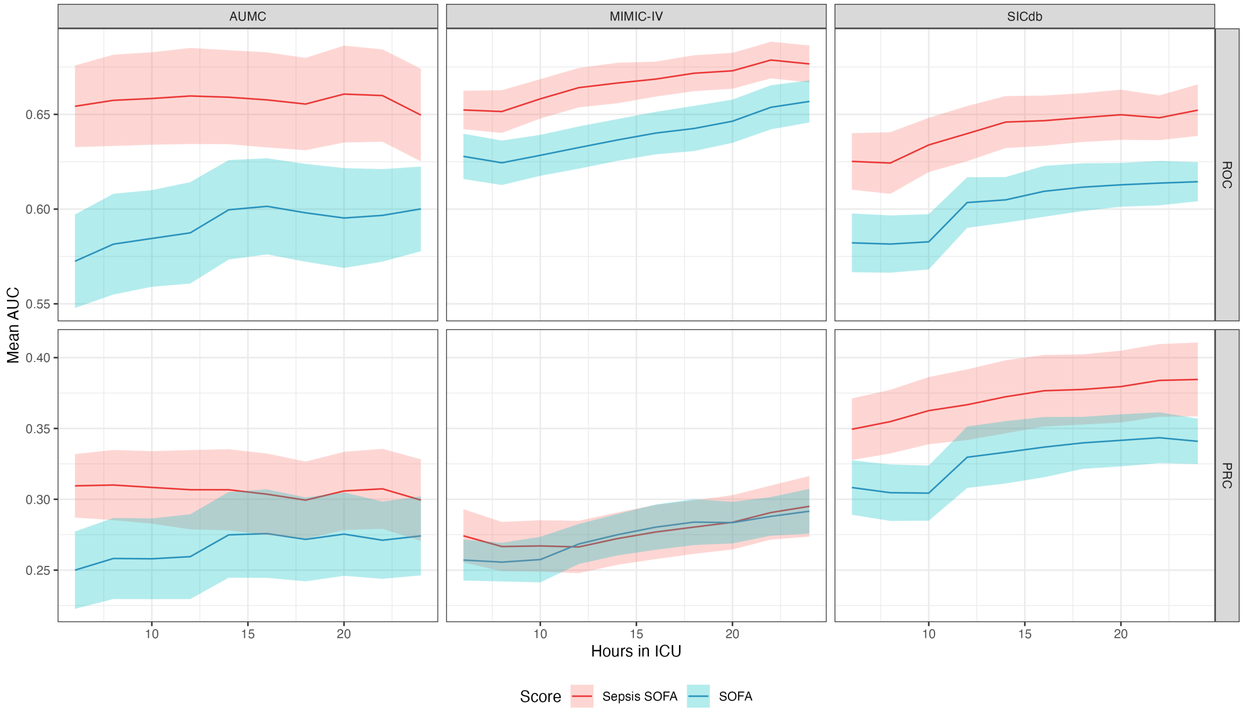

eFigure 3: Over-time performance of the data-driven Sepsis SOFA and SOFA scores, in the cohort of patients who received antibiotics for at least 4 consecutive days. The two scores are evaluated in terms of area under receiver operator characteristic (AUROC) and area under precision recall (AUPRC) for predicting mortality, during the first day of ICU stay, in time steps of 2 hours, with the metabolic component of Sepsis SOFA not included. The 95% confidence intervals for the areas under the curve, obtained using bootstrap, are plotted in every subplot. The new score still outperforms SOFA in each metric, time point and dataset.

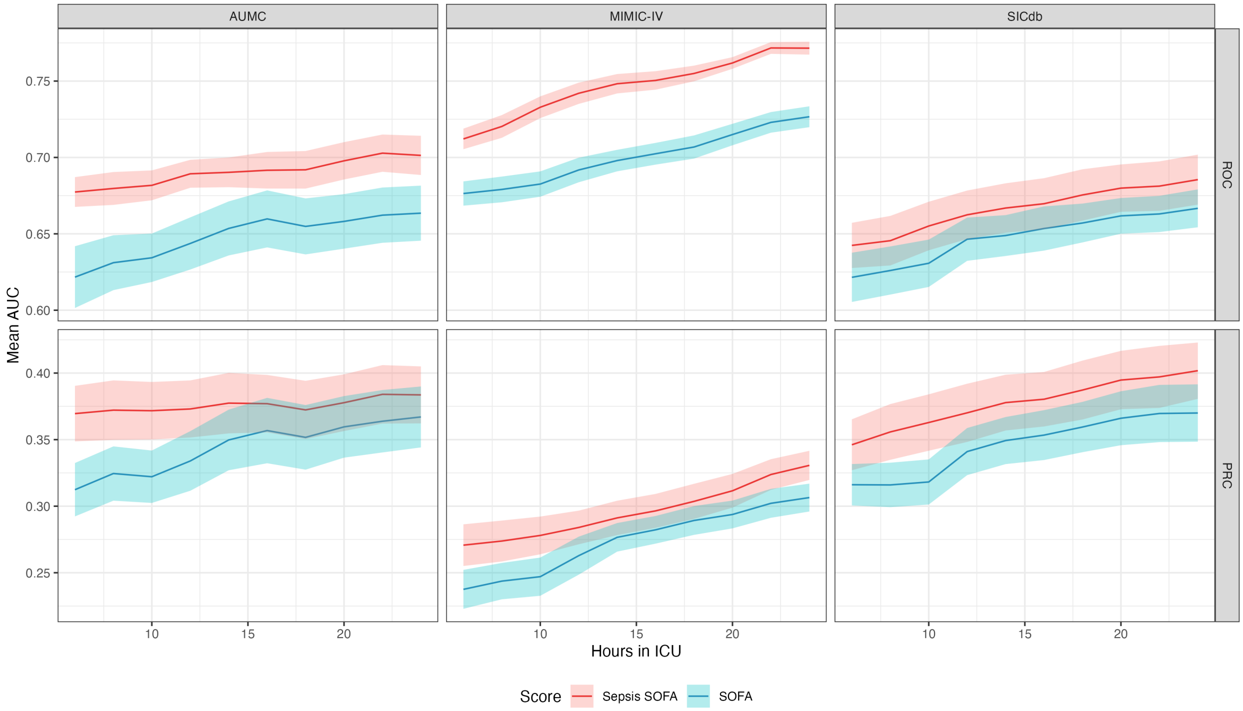

eFigure 4: Over-time performance of the data-driven Sepsis SOFA for low- and middle-income countries and SOFA. The two scores are evaluated in terms of area under receiver operator characteristic (AUROC) and area under precision recall (AUPRC) for predicting mortality, during the first day of ICU stay, in time steps of 2 hours, with the metabolic component of the Sepsis SOFA score based on bicarbonate, and the respiratory component on SpO2/FiO2 ratio. The 95% confidence intervals for the areas under the curve, obtained using bootstrap, are plotted in every subplot.

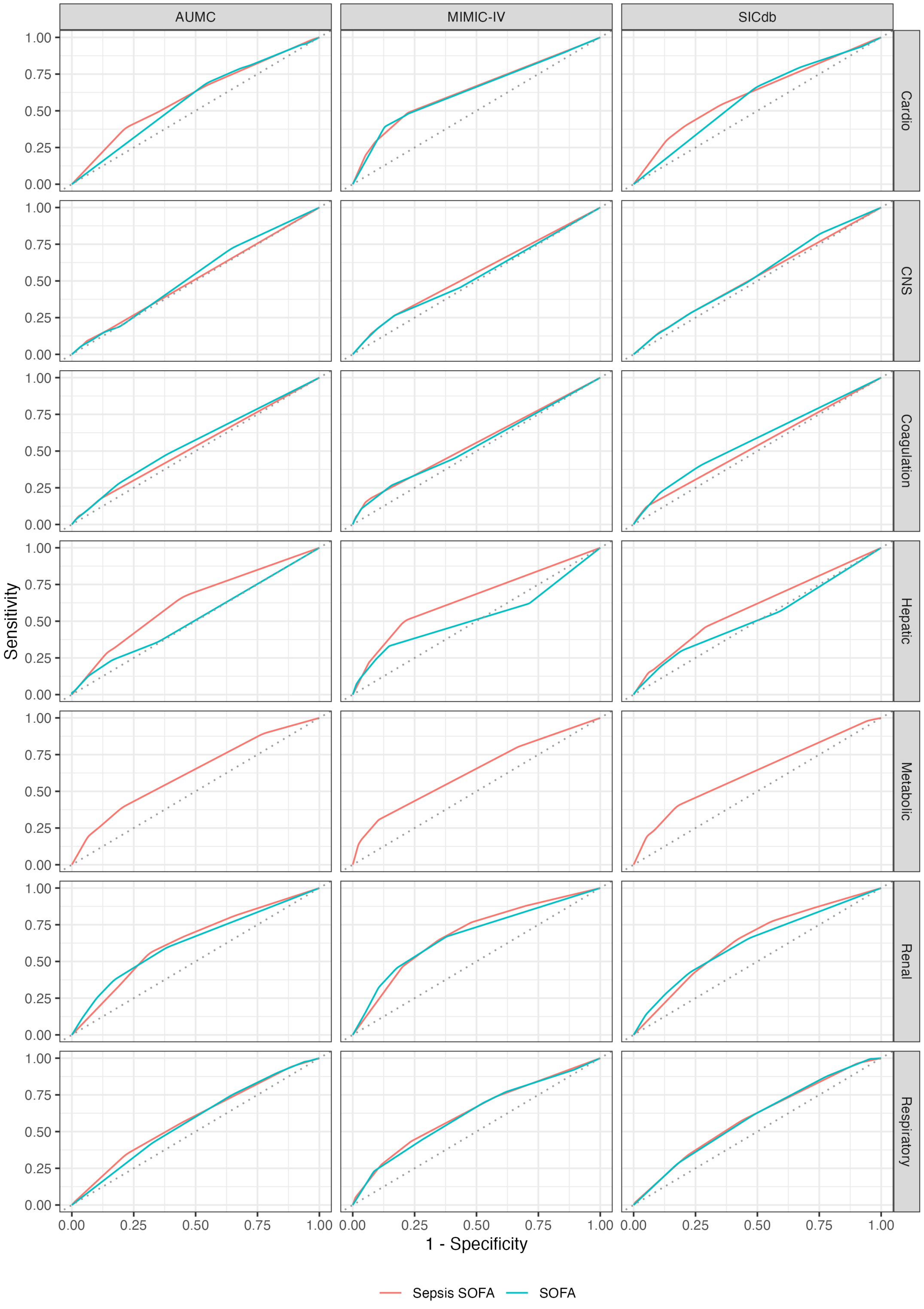

eFigure 5: Performance of the data-driven Sepsis SOFA and SOFA scores at 24 hours into ICU. Each component of Sepsis SOFA is compared to the corresponding component of SOFA, by plotting the receiver operator characteristic (ROC) curve at 24 hours into ICU stay. Each row of the figure corresponds to an organ failure category (cardiovascular, coagulation, hepatic, metabolic, renal, respiratory) and each row corresponds to a dataset. Sepsis SOFA outperforms SOFA in almost every component and dataset.

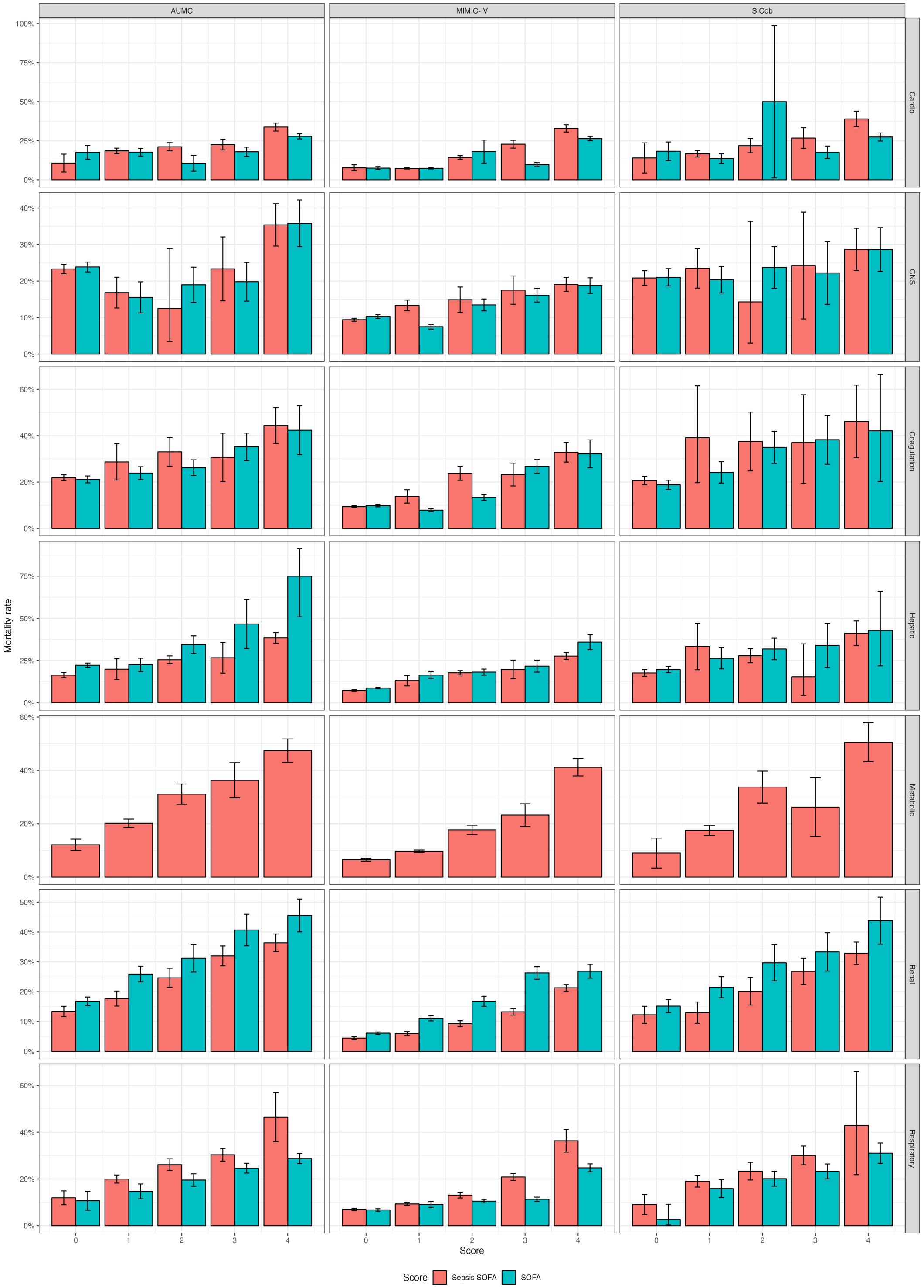

eFigure 6: Mortality barplots for each component and dataset. For each organ failure component, we divide the study cohort into groups of patients who had a Sepsis SOFA score of 0, 1, 2, 3, or 4 at 24 hours into ICU, and calculate the mortality rate in each group. The same was repeated for all the values and components of the SOFA score. The mortality rate for each group, dataset and component are presented as barplots, where each row of the figure corresponds to an organ failure category (cardiovascular, coagulation, hepatic, metabolic, renal, respiratory) and each row corresponds to a dataset.

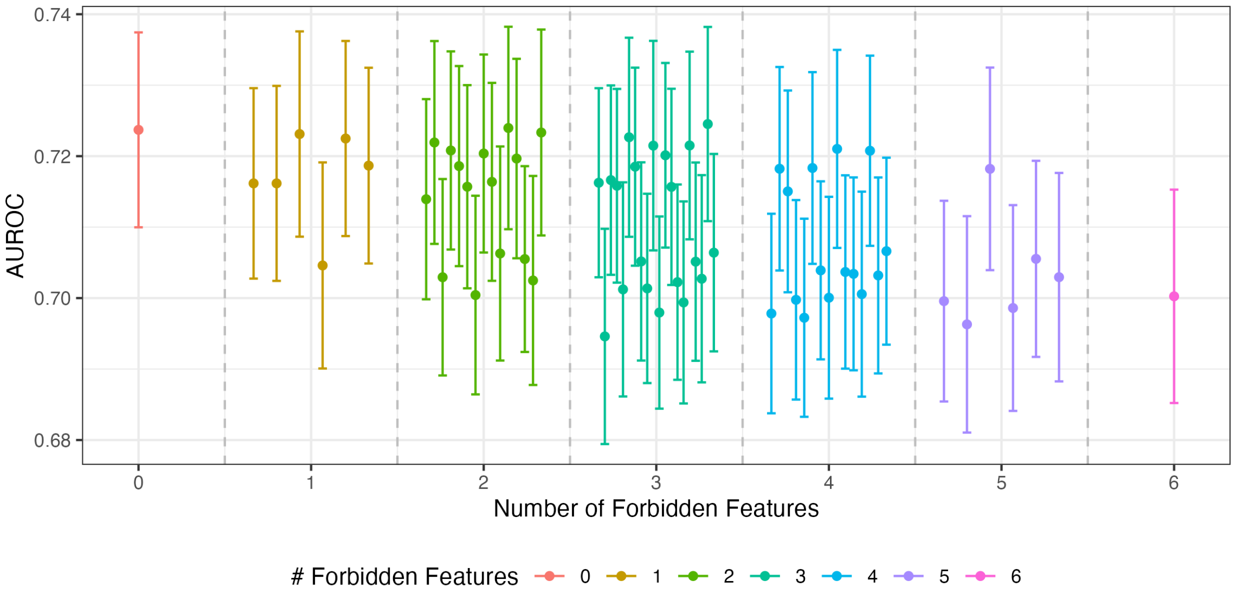

eFigure 7: Performance of predictive scores in the ablation analysis. The average AUROC at 24 hours into ICU stay on the validation set is reported, together with its 95% confidence interval. The score with 0 forbidden features is the optimal score presented in Table 2. For each of the six domains (excluding neurological), we reconstructed a predictive score with the most predictive feature forbidden (yielding six scores with 1 forbidden feature). Then, we listed any combination of 2 most predictive features, and constructed scores which did not use these features. This was continued until, finally, we constructed a score that was forbidden to use any of the features appearing in the original optimal score in Table 2. The figure indicates that even removing several of the optimal features still yields a relatively good predictive performance of the score.

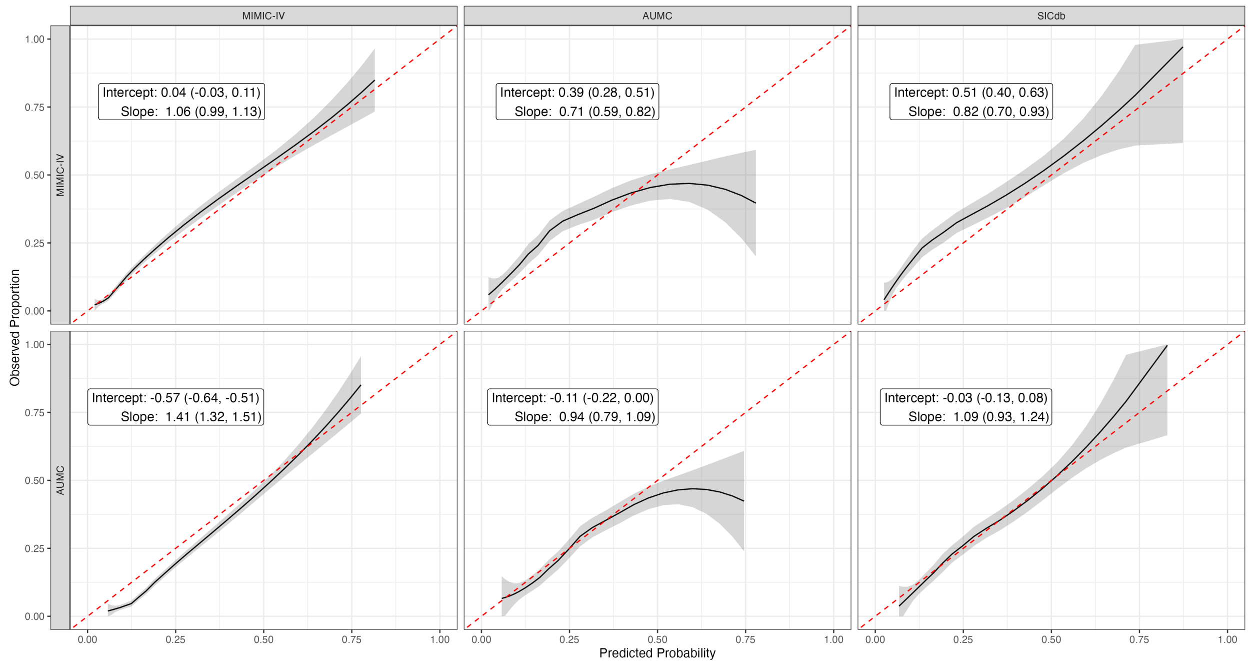

eFigure 8: Calibration curves of Sepsis SOFA across development (vertical) and validation (horizontal) datasets. For each development cohort (MIMIC-IV, AUMC), we compute the Sepsis SOFA values. Based on these, we fit a logistic regression model (death Sepsis SOFA + Intercept), and the fitted probabilities are used as predicted probabilities. The fitted probabilities are then assessed for calibration on the validation cohorts (MIMIC-IV, AUMC, SICdb).

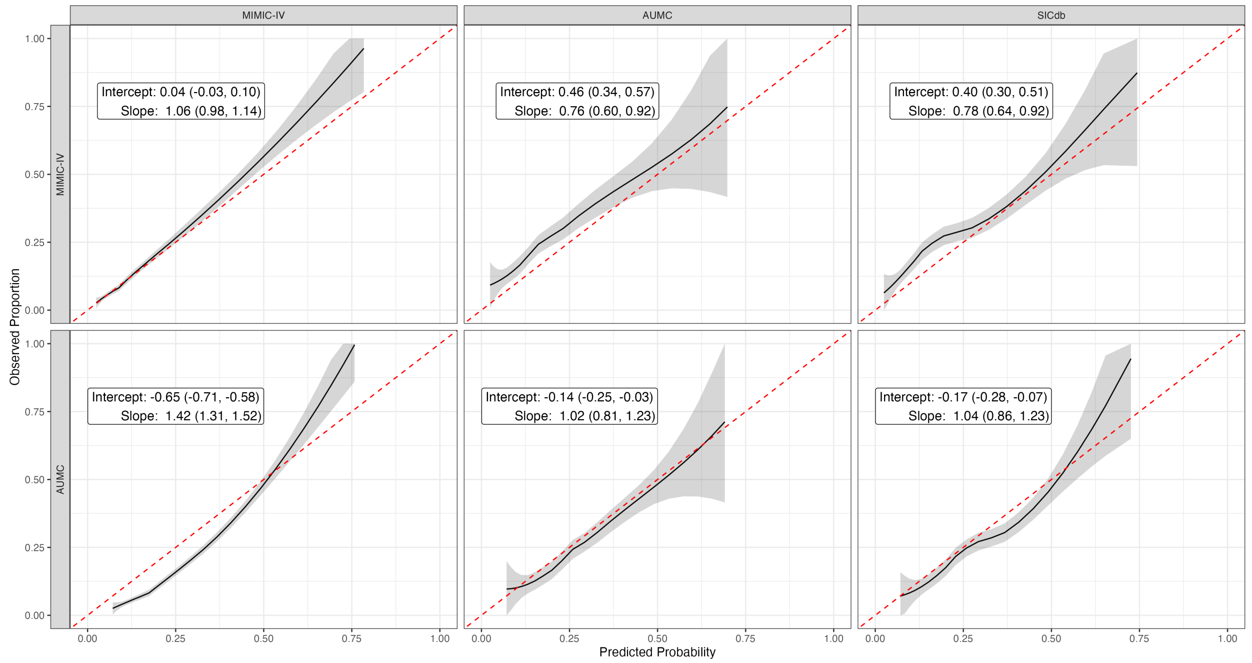

eFigure 9: Calibration curves of SOFA across development (vertical) and validation (horizontal) datasets. For each development cohort (MIMIC-IV, AUMC), we compute the SOFA values. Based on these, we fit a logistic regression model (death SOFA + Intercept), and the fitted probabilities are used as the predicted probabilities. The fitted probabilities are then assessed for calibration on the validation cohorts (MIMIC-IV, AUMC, SICdb).
