# Supplementary material for: A framework and analytical exploration for a data-driven update of the Sequential Organ Failure Assessment (SOFA) score in sepsis
Source: Crit Care Resusc. 2025 Mar 14;27(1):100105. doi: 10.1016/j.ccrj.2025.100105 (PMC11952785; doi:10.1016/j.ccrj.2025.100105)
Supplement: Multimedia component 5 [file mmc5.pdf]

eAppendix: AUC maximization

This appendix describes the AUC maximization procedure that was used to construct the data-driven update of the SOFA score.

Step 1: Feature selection

The feature selection procedure worked as follows. For every feature, the worst value in the first 24 hours of ICU stay was determined for every patient. Based on these values, for each feature the area under receiver operator characteristic (AUROC) for predicting mortality was computed. Each feature was then categorized into one of the following categories: cardio, CNS, coagulation, liver, renal, respiratory and metabolic.

Step 2: Threshold selection

In the second step, for each feature, we determined its clinically relevant range, together with an increase that would be considered clinically significant. For example, the relevant range for blood urea nitrogen was taken to be between 15 and 100 mg/dl, where an increase of 5 mg/dl was deemed significant. Then, for each score component we selected the 4 thresholds and a feature that maximized the AUROC. After this, the score was improved in an iterative fashion, described in the following procedure:

Algorithm 1: AUC maximization

score $\leftarrow$ initial score ▷ Initialize the score
$\delta \leftarrow 0.002$ ▷ Minimal relevant AUROC improvement in each step
continue $\leftarrow$ true ▷ Continuation variable
while *continue = true* **do**
    continue $\leftarrow$ false
    for *each component* $\mathcal{C}$ **do**
        for *each feature* $\mathcal{F}$ *in component* $\mathcal{C}$ **do**
            find optimal t_1, t_2, t_3, t_4 for $\mathcal{F}$ ▷ Find best thresholds **if** *AUROC improved by* $\geq \delta$ **then**
                update component $\mathcal{C}$ with t_1, t_2, t_3, t_4 and feature $\mathcal{F}$
                continue $\leftarrow$ true ▷ Procedure continues
return *score*

In words, we iterated over each component of the new score. We checked whether the AUROC of the score could be improved (by at least $\delta = 0.2\%$) by a new selection of thresholds and features, while keeping all other components fixed. After each step, we moved onto the next component and repeated the process, until the score could no longer be improved.

Performance Metrics and Additional Analyses

The two metrics used for performance evaluation in the paper were the area under the receiver operating characteristic curve (AUROC) and the precision-recall curve (AUPRC).

The receiver operating characteristic (ROC) curve shows the relationship between the true positive rate (also known as sensitivity or recall) and the false positive rate (1-specificity) for every possible cut-off. The ROC is a graph with the x-axis showing the false positive rate, defined as:

$$\text{False Positive Rate} = \frac{\text{False Positives}}{\text{False Positives} + \text{True Negatives}},$$

and the y-axis showing the true positive rate (sensitivity), defined as:

$$\text{True Positive Rate (Sensitivity)} = \frac{\text{True Positives}}{\text{True Positives} + \text{False Negatives}}.$$

The precision-recall curve (PRC) shows the relationship between precision (positive predictive value) and recall (sensitivity) for every possible cut-off. The PRC is a graph with the x-axis showing recall (sensitivity) and the y-axis showing precision (positive predictive value), defined as:

$$\text{Precision (Positive Predictive Value)} = \frac{\text{True Positives}}{\text{True Positives} + \text{False Positives}}.$$
