# Supplementary material for: A framework and analytical exploration for a data-driven update of the Sequential Organ Failure Assessment (SOFA) score in sepsis
Source: Crit Care Resusc. 2025 Mar 14;27(1):100105. doi: 10.1016/j.ccrj.2025.100105 (PMC11952785; doi:10.1016/j.ccrj.2025.100105)
Supplement: Multimedia component 6 [file mmc6.pdf]

eAppendix: Vasopressor Adjusted MAP

This appendix gives more detail on how the we converted vasopressor dosages into norepinephrine equivalents (NEQ), how we adjusted the mean arterial pressure (MAP) by NEQ, followed by a worked example.

Norepinephrine equivalents

To convert vasopressor amounts into equivalents of norepinephrine, we used the conversion rates shown in Table 1.

Vasopressor drug	Rate	Norepinephrine Equivalents
Epinephrine	0.1 mcg/kg/min	0.1 mcg/kg/min
Norepinephrine	0.1 mcg/kg/min	0.1 mcg/kg/min
Dopamine	15 mcg/kg/min	0.1 mcg/kg/min
Vasopressin	0.04 U/min	0.1 mcg/kg/min
Phenylephrine	1 mcg/kg/min	0.1 mcg/kg/min

Table 1: Conversion table for converting epinephrine, dopamine, vasopressin and phenylephrine to equivalents of norepinephrine.

Vasopressor Adjusted MAP

After the computing the norepinephrine equivalents of each vasopressor treatment period, we adjusted the MAP value by subtracting the value of norepinephrine equivalents (NEQ) multiplied by a factor β, that is

$$\text{Vasopressor Adjusted MAP } (\beta) = \text{MAP} - \beta * \text{NEQ}.$$

Multiple different values of the β parameter were considered, namely $\beta \in \{10, 50, 100, 200\}$. The AUC-maximization procedure was left to choose the optimal value of β in terms of predictive performance for mortality, within the suspected infection cohort.
For concreteness, we show several worked examples.

Example 1. If a patient is receiving 0.2 mcg/kg/min of norepinephrine and has a MAP of 65 mmHg, then the Vasopressor Adjusted MAP equals

$$\text{MAP}(200) = 65 \text{ mmHg} - 200 * 0.2 \text{ mcg/kg/min} = 25.$$

Example 2. If a patient is receiving 30 mcg/kg/min of dopamine and has a MAP of 65 mmHg, then the Vasopressor Adjusted MAP equals

$$\text{MAP}(200) = 65 \text{ mmHg} - 200 * (30 \text{ mcg/kg/min } * \frac{0.1}{15}) = 25.$$

Example 3. If a patient is receiving 0.04 U/min of vasopressin and 15 mcg/kg/min of dopamine and has a MAP of 65 mmHg, then the Vasopressor Adjusted MAP equals

$$\text{MAP}(200) = 65 \text{ mmHg} - 200 * (\underbrace{15 \text{ mcg/kg/min } * \frac{0.1}{15}}_{\text{dopamine to NEQ}} + \underbrace{0.04 \text{ U/min } * \frac{0.1}{0.04}}_{\text{vasopressin to NEQ}}) = 25.$$

Example 4. If a patient is receiving 0.02 U/min of vasopressin and 1 mcg/kg/min of phenylephrine and has a MAP of 65 mmHg, then the Vasopressor Adjusted MAP equals

$$\text{MAP}(200) = 65 \text{ mmHg} - 200 * (\underbrace{1 \text{ mcg/kg/min } * \frac{0.1}{1}}_{\text{phenylephrine to NEQ}} + \underbrace{0.02 \text{ U/min } * \frac{0.1}{0.04}}_{\text{vasopressin to NEQ}}) = 35.$$
